# Making Every Contact Count: health professionals’ experiences of integrating conversations about Snacktivity to promote physical activity within routine consultations – a qualitative study

**DOI:** 10.1136/bmjopen-2024-085233

**Published:** 2024-10-22

**Authors:** Matthew Krouwel, Sheila Greenfield, James P Sanders, Kajal Gokal, Anna Chalkley, Ryan A Griffin, Helen Parretti, Kate Jolly, Magdalena Skrybant, Stuart Biddle, Colin Greaves, Dale W Esliger, Lauren B Sherar, Charlotte Edwardson, Thomas Yates, Ralph Maddison, Emma Frew, Nanette Mutrie, Natalie Ives, Sarah Tearne, Amanda J Daley

**Affiliations:** 1 Centre for Lifestyle Medicine and Behaviour, School of Sport, Exercise and Health Sciences, Loughborough University, Loughborough, UK; 2 Institute of Applied Health Research, University of Birmingham, Birmingham, UK; 3 Faculty of Life Sciences and Health Studies, University of Bradford, Bradford, UK; 4 Centre for Applied Education Research, Wolfson Centre for Applied Health Research, Bradford Royal Infirmary, Bradford, UK; 5 BCTU, University of Birmingham, Birmingham, UK; 6 Norwich Medical School, University of East Anglia, Norwich, UK; 7 University of Southern Queensland, Toowoomba, Queensland, Australia; 8 School of Sport, Exercise and Rehabilitation Sciences, University of Birmingham, Birmingham, UK; 9 School of Sport, Exercise and Health Sciences, Loughborough University, Loughborough, UK; 10 Diabetes Research Centre, College of Life Sciences, University of Leicester and NIHR Leicester Biomedical Research Centre, Leicester, UK; 11 Institute for Physical Activity and Nutrition, Deakin University, Melbourne, UK; 12 Physical Activity for Health Research Centre, University of Edinburgh, Edinburgh, UK

**Keywords:** QUALITATIVE RESEARCH, Health Education, MEDICAL EDUCATION & TRAINING, Primary Prevention

## Abstract

**Abstract:**

**Objective:**

Helping people to change their health behaviours is becoming a greater feature within the role of health professionals, including through whole system initiatives such as Making Every Contact Count. Health services provide an ideal setting to routinely promote health behaviours, including physical activity. Snacktivity is a novel approach that promotes small bouts of physical activity (activity snacks) throughout the day. This study explored health professionals’ initial experiences of delivering a Snacktivity intervention to promote physical activity within routine health consultations. A further aim was to investigate health professionals’ ability/fidelity in delivering the Snacktivity intervention to their patients.

**Design:**

Semistructured interviews (n=11) and audio recording of consultations (n=46).

**Setting and participants:**

Healthcare professionals from a variety of specialisms who delivered the Snacktivity intervention within patient consultations.

**Results:**

Analyses revealed two higher-level themes of interest: (1) health professionals’ conceptualisation of Snacktivity (subthemes: observations/reflections about patients’ understanding, engagement and enthusiasm for delivering the Snacktivity intervention) and (2) health professionals’ understanding of Snacktivity and experience in delivering the intervention (subthemes: delivering Snacktivity; limitations, challenges and possible improvements). Consultation audio recordings demonstrated health professionals delivered the Snacktivity intervention with high levels of fidelity. Health professionals were proficient and supportive of delivering the Snacktivity intervention within consultations although practical barriers to implementation such as time constraints were raised, and confidence in doing so was mixed.

**Conclusions:**

Health professionals were proficient and supportive of delivering the Snacktivity intervention within consultations. The primary barrier to implementation was the time to deliver it, however, gaining greater experience in the intervention and improving behaviour change counselling skills may reduce this barrier.

**Trial registration number:**

ISRCTN64851242.

**STRENGTHS AND LIMITATIONS OF THIS STUDY**

- Convenient samples of health professionals and patients/participants were recruited.
- A modest (small) number of health professionals were interviewed but audio recordings from a large number of consultations were included.
- This is the first study to explore the merits of a whole systems approach to health professionals promoting the Snacktivity intervention within consultations.
- The initial views of health professionals working in a wide range of health settings were captured.

## Introduction

 Chronic diseases are the leading global cause of disability and premature mortality, with physical inactivity contributing substantially to the burden of disease.^1^ The importance of supporting people to change their health behaviours is becoming more recognised within the role of healthcare professionals (HCPs). Health services provide an ideal context in which to promote physical activity because of the potential to reach across populations at scale. There is also evidence that brief opportunistic health behaviour interventions have the potential for effectiveness.^2 3^ The example of the Making Every Contact Count (MECC) initiative, adopted in several countries (eg, England, Wales and Ireland), aims to enable all HCPs to routinely discuss and deliver brief health behaviour change interventions within their interactions and consultations with the public.^4^ Other whole system initiatives with similar aims have also been developed, such as Moving Medicine, Moving Healthcare Professionals Programmes and Exercise Is Medicine.^5 6^

Those who are regularly engaging with HCPs have, or are at risk of, developing health conditions and are more likely to be inactive. Furthermore, one in four people would be more active if advised by an HCP, and therefore, they play a critical role in supporting people to be more physically active.^5^ The success of approaches such as MECC is heavily dependent on routine implementation across health services by HCPs. There may be varied barriers and enablers to delivering health behaviour change interventions within consultations, particularly for promoting physical activity, a behaviour that requires more than cursory conversation by HCPs to facilitate change.

While the potential for HCPs to reduce the prevalence of health behaviour disease risk factors might be intuitively appealing (through approaches such as MECC), in practice, it is complex to achieve. It is important that HCPs feel comfortable delivering health behaviour interventions to their patients, to support them to make changes that could improve their health, however, evidence has highlighted that many HCPs do not feel confident doing so.^7^ Furthermore, research has reported that HCPs can find these conversations difficult, frustrating and some have questioned whether they are worthwhile, particularly when raising the topic with patients who are consulting for reasons unrelated to health behaviours.^8^,^11^ Other evidence has found HCPs to be supportive of MECC approaches, but that they have concerns about their capability and capacity to deliver them.^12^ Studies suggest that only ~50% of HCPs in the UK deliver brief interventions, even when they perceive patients would benefit from the intervention.^11^ Patients also need to be receptive to having such conversations in consultations and then be willing to act, although resistance by patients to these types of conversations has been reported.^13^

### Snacktivity and MECC

The inclusion of conversations about physical activity within health consultations may be beneficial because many adults do not participate in sufficient amounts of physical activity.^5^ The Snacktivity programme of research (https://www.fundingawards.nihr.ac.uk/award/RP-PG-0618-20008) aims to explore the health benefits of participation in short ‘snacks’ of physical activity throughout the day and the delivery of the Snacktivity intervention within health settings. Activity snacks can be defined as bouts of at least moderate intensity physical activity that typically lasts between 2 and 5 min, consistent with recent guidance that has stated all physical activity is important for health.^14^ Snacktivity seeks to promote both aerobic and resistance/strength-based physical activity, through HCPs raising and discussing physical activity and Snacktivity within consultations. Details regarding Snacktivity have been published previously and evidence suggests that the public appreciates the Snacktivity approach to physical activity and views it as worthwhile.^15^,^17^

Using semistructured interviews, the purpose of this study was to explore HCPs initial views of delivering a Snacktivity intervention within routine health consultations and to understand their views of doing this within the context of the MECC initiative. There is a lack of research on the implementation of health system-wide initiatives to promote physical activity within the practice of HCPs, particularly in specialisms of health/medicine that are not typically involved in delivering lifestyle behaviour interventions. This study aimed to explore the experiences of a variety of HCPs in delivering the Snacktivity intervention within different health contexts. A further aim was to assess the fidelity of delivery by HCPs in implementing the Snacktivity intervention after completing the intervention delivery training module.

## Materials and methods

### Study design and context

This research was conducted between January 2021 and December 2022. HCPs (podiatrists, hospital dentists, dietitians, physiotherapists, practice nurses, healthcare assistants and general practitioners/family doctors) were recruited to deliver the Snacktivity intervention in two studies (study A and study B), to test the feasibility of delivering the intervention within health services.^18 19^ Data from the studies have been combined and are reported here as a single study. Study A took place within a community health setting with all consultations delivered by podiatrists (uncontrolled trial) where patients who had consented to the trial received the Snacktivity intervention within their podiatry consultation.^18^ Study B took place within primary care, community health and public health settings as part of a feasibility randomised controlled trial, where HCPs (listed above) delivered the intervention to those allocated to the Snacktivity intervention group.^19^ Participants in both studies provided written informed consent to their consultations being audio recorded by their healthcare provider. In study A, HCPs obtained written informed consent for the consultation to be audio recorded, while in study B, this consent was obtained by the research team as part of the overall consent procedures for the trial. Additionally, some HCPs agreed to participate in a semistructured interview to explore their experiences of delivering the Snacktivity intervention to their patients within consultations.

### Snacktivity intervention

The Snacktivity intervention has been described in detail elsewhere.^18 19^ HCPs promoted the behavioural goal of encouraging patients/participants to work towards accumulating ≥150 min of moderate to vigorous physical activity per week via Snacktivity throughout the day. The intervention was designed as a brief intervention (~5–7 min) where HCPs raised the topic of Snacktivity, explained the purpose and benefits of Snacktivity and then encouraged individuals to engage with the Snacktivity technology (Fitbit device linked with a bespoke mobile phone app: ‘SnackApp’). This technology allows people to receive support and feedback to encourage participation in Snacktivity throughout each day. HCPs were not involved in the setup or distribution of the Snacktivity intervention technology, other than to highlight in consultations that this resource was available to their patients/participants free of charge, to facilitate their participation. All HCPs attended a 1-hour training session, led by the research team before delivering the intervention. In study A, the HCP training was delivered face to face in a group setting. In line with COVD-19 restrictions at the time, the training for study B was conducted online in groups of 2–3 HCPs. HCPs were asked to follow a 12-point consultation checklist to facilitate consistent delivery of the Snacktivity intervention.

### Recruitment of HCPs

Towards the end of the studies, a convenience sample of HCPs who had delivered the Snacktivity intervention in consultations were invited by a researcher, through email or phone, to take part in a semistructured interview about their experiences of promoting Snacktivity within consultations. Those interested in participating in an interview were given the opportunity to ask questions about the study and provided written informed consent before being interviewed.

### Interview process and topic of interest

The semistructured interviews were conducted by two researchers (AC for study A and MK for study B). AC is a female postdoctoral qualitative researcher with 5 years of experience and an interest in health behaviours. AC was known to the HCPs as part of the trial component of this research and had a high level of knowledge of the Snacktivity programme and the expectations of the HCP. MK is a male postdoctoral freelance researcher with over 5 years of experience in qualitative research who had no prior contact with the HCPs, or any prior introduction to them and at the time of the interviews had only been supplied with a brief overview of the role of the HCPs in the Snacktivity programme. The interview topic guide (online supplemental appendix 1) aimed to explore the views of HCPs about delivering and integrating the Snacktivity intervention within healthcare consultations. The interview schedule consisted of a series of predetermined, topic-orientated and predominantly open-ended questions and provided the opportunity for HCPs to talk more widely about their experiences while remaining relevant to the topic of interest.^20^ The topic guide was not piloted prior to the interviews.

Interviews were conducted by telephone or video call, depending on the preferences of HCPs and recorded using encrypted audio recorders. Brief field notes were made after each interview capturing any relevant contextual information and reflections on discussions, however, formal records were not kept of participants’ location or the presence of other people during the interview. Saturation was not the aim of this research as the number of HCPs was limited by the number who had delivered the Snacktivity intervention. As such, an information power model^21^ was used which encouraged interviewing every participant who consented. A diverse team was used to enhance the trustworthiness of the research,^22^ which included two experienced postdoctoral qualitative researchers (MK and AC), who were employed to conduct this research; a specialist in behavioural medicine (AD) and a medical sociology (SG) who designed the research (along with the wider research team) and supported data analyses. All recordings were transcribed by a commercial company, which had a confidentiality agreement in place with Loughborough University. Transcriptions were not returned to participants prior to analysis.

### Fidelity of HPCs in delivering the Snacktivity intervention: audio recordings

A range of HCPs (podiatrists, hospital dentists, dietitians, physiotherapists, practice nurses, healthcare assistants and general practitioners) recorded consultations with consenting patients using an encrypted audio recording device and fidelity of delivery was rated using an intervention checklist (online supplemental appendix 2). Only the segment of the consultation that related to delivery of the Snacktivity intervention was audio recorded, not the whole clinical consultation. Audio recordings of the interactions were transcribed by the same commercial company used for the interview transcripts.

### Patient and public involvement

This study report forms part of a larger programme of work where a public advisory group consisting of 10 patient members contribute their experience to the development and scope of each study within the programme. In the current study, the public advisory group was involved in providing feedback on all patient-facing documentation and materials.

### Data analysis of semistructured interviews with HCPs

As there were some differences between the settings in which the data were gathered, and by different interviewers, the data sets from study A and study B were analysed independently and then combined. It was considered that this approach would highlight any substantive differences between the data sets and guide the decision as to whether the data sets could be subsequently combined. The analysis of the data sets followed the same procedure. Transcriptions were analysed using inductive thematic analysis to identify themes.^23^ Data management and analysis were facilitated by the NVivo V.12 software package. The first three HCP interview recordings were coded (MK) and initial themes were identified, critically discussed and agreed within the team (MK and SG), adjustments were made to theme titles/definitions and the remaining data were coded.^24^ Any proposed changes to the themes thereafter were discussed and agreed within the wider team (AD, MK and SG). Participant feedback on themes and findings was not part of this study.

### Data analysis of consultation audio recordings to assess fidelity

Transcripts of consultations were imported into the NVivo V.12 software package. Snacktivity intervention fidelity was assessed by one rater (MK) who compared the discourse content of the consultation audio recordings between HCPs and patients against the intervention checklist. Because the intervention is intended to be integrated into a routine consultation in a natural/routine way, HCPs were allowed to use their own phrasing to deliver the intervention rather than be directed to read the 12 checklist items out verbatim to participants. This resulted in the assessor (MK) making a judgement as to whether or not a checkpoint item had been covered sufficiently. Additionally, an approach was taken in which if any elements in the item checklist description were mentioned then it was considered that the item had been raised and one point was awarded. For example, the first item on the checklist refers to mentioning the importance of physical activity for physical, mental and muscular health, in this case, if any one of these was raised the item was considered to have been mentioned. A total summary score for each HCP was calculated (yielding a score from 0 to 12).

## Results

### Interviews with HCPs

11 interviews with HCPs were completed with an average duration of 29.5 min (range 19–43 min). Of the HCPs interviewed, five were podiatrists, three hospital dentists and three physiotherapists (see table 1). The two analyses identified three themes each (see table 2), with only minor differences, which were subsequently combined into a single analysis. The final themes consisted of HCPs’ conceptualisation of Snacktivity, experience in the non-intervention elements of being in a trial and HCPs’ experiences of delivering the Snacktivity intervention. Of these themes, the experience in the non-intervention elements of being in the trial was not considered relevant to the aims of this study as it identifies information related to the conduct of the trials and is in online supplemental appendix 3.

**Table 1:** Demographics of HCPs interviewed

ID number	Male/female	Job role	Snacktivity consultations delivered
Study A			
WP2-HCP001	Male	Podiatrist	3
WP2-HCP002	Male	Podiatrist	4
WP2-HCP003	Male	Podiatrist	2
WP2-HCP004	Female	Podiatrist	3
WP2-HCP005	Female	Podiatrist	4
Study B			
WP3-HCP001	Male	Physiotherapist	3
WP3-HCP007	Female	Dentist	2
WP3-HCP008	Male	Physiotherapist	1
WP3-HCP009	Female	Dentist	2
WP3-HCP010	Female	Dentist	2
WP3-HCP011	Male	Physiotherapist	2

HCPshealthcare professionals

**Table 2:** Independently identified themes and combination of themes process

Study A main themes	Study A subthemes	Study B main themes	Study B subthemes	Combined main themes	Combined subthemes
Observations from HCPs	Participants’ understanding	HCPs conceptualisation of Snacktivity	Patients’ understanding of Snacktivity	HCPs conceptualisation of Snacktivity (its meaning and purpose)	Snacktivity as it is understood by the HCP
	Participants’ enthusiasm/morale				Observations and reflections about patients’ understanding, engagement and enthusiasm.
HCPs experience outside of pure delivery factors. Specifically, this refers to anything which is not a front-line Snacktivity operation, for example, elements of managing the trial (recruitment, administration) and being trained in delivering Snacktivity	Training	Experience in delivering the Snacktivity intervention	Training	Experiences of the non-delivery elements of being in a clinical trial	Reflections on the training experience
	Engagement with trial		Engagement with research		Motivation and experience in engagement with the trial
			Delivery	Perspectives about delivering the Snacktivity intervention in consultations	Delivering Snacktivity
Experience in delivering the Snacktivity intervention from the perspective of the HCP	Limitations, barriers and improvements	Experience in being an HCP in the Snacktivity trial	Challenges		Limitations, challenges and possible improvements

HCPshealthcare professionals

### Health professionals’ conceptualisation of Snacktivity

Substantial discourse was apparent in terms of what was understood by HCPs regarding the term ‘Snacktivity’, both in relation to its meaning and purpose and their observations of patients’ understanding of this word and concept.

#### Snacktivity as understood by HCPs

HCPs appeared to understand the Snacktivity concept and what this approach to promoting physical activity aimed to achieve. In addition, some HCPs had previously encountered versions of the Snacktivity approach to promoting physical activity.

I had heard about it before, not actually the name Snacktivity, but the fact doing little bursts of exercise may be better than doing … if you don’t have time, than doing the … like an hour or thirty minutes per day. (Female dentist—WP3 HCP007)

Although, for at least one HCP, it was a new way of approaching physical activity.

It seems difficult relating, let’s say going for a run or going for something where you definitely get a bit sweaty, your heart rate goes up, versus just being physically more active during the day, it doesn’t necessarily seem to correlate. (Female dentist—WP3 HCP009)

Some HCPs had thoughts about who and which patients were most likely to benefit from Snacktivity. There was also the perception that Snacktivity had the potential to alter patients’ attitudes towards physical activity.

This could be more used for a lot of people really, you know people who are busy or not in great shape physically perhaps, maybe more elderly as well, so it might be useful for many people. (Male physiotherapist—WP3 HCP011)

I think that was a useful concept to bring in and try and break down that barrier that physical activity is always putting your gym kit on and actually going to the gym or physically going to do sport or something like that. So that was a useful concept. (Male physiotherapist—WP3 HCP008)

Another HCP was aware that people might be inhibited by the unusualness of the behaviour.

…jumping on the spot or I don’t know, doing squats while you’re in the kitchen or something like that, that might just be a little bit awkward for people perhaps to get to start off with. (Female dentist—WP3 HCP010)

However, other HCPs were more enthusiastic, perceiving that the name conveyed the approach effectively.

I really believe in the approach, even down to the name, I think it’s absolute genius! (both laugh) Snacktivity, it’s just genius! Because it encapsulates it so well and it gives the participant something to hang on to… (Male podiatrist—WP2 HCP02)

Some potential problems were suggested in relation to the specific nature of the Snacktivity consultation, with two physiotherapists highlighting that talking about Snacktivity could detract from the specific physiotherapeutic focus of the work they were trying to achieve with patients. Another physiotherapist was concerned that they may need to prescribe specific sustained exercise, rather than small and repeated bouts, which could result in conveying a conflicting message to patients. The dentists were cautious about how the introduction of the topic of physical activity would work in a dental health environment.

And I guess I was interested to see how it would work in dentistry, because I think we were a little bit apprehensive, if I wanted to be honest with you, about how we can link this with dental health and physical activity… (Female dentist—WP3 HCP010)

I think … yeah, again as it’s not part of your day to day as a dentist, I’m not saying that it shouldn’t be… (Female dentist-WP3 HCP009)

However, once dentists began to introduce Snacktivity into consultations these concerns appeared to have been reduced by patients’ willingness to discuss the topic.

And patients did seem interested when we spoke to them about it … the majority … most of them did. (Female dentist—WP3 HCP007)

#### Observations and reflections on patients’ understanding, engagement and enthusiasm

The HCPs made various observations regarding their patients who were participating in this research, which primarily focused on participants’ engagement with, and understanding of, Snacktivity. It was expressed that patients were enthusiastic about Snacktivity, however, there was an awareness that, for some, the challenges of using the supporting Snacktivity technology reduced this initial enthusiasm, which in one case resulted in withdrawal from the study.

The next time I saw him, he’d said, oh I’ve pulled out of that Snacktivity thing and I sent everything back. And I was like, why? And he just says, oh I couldn’t get to grips with the watch and the app and … it just… it all flustered me. (Male podiatrist-WP2 HCP003)

HCPs appeared to feel that patients had a good understanding of Snacktivity, with a couple highlighting that their patients had no questions about it and one cited an instance where the study materials had sufficiently prepared the patient so that no further explanation was required. Despite this, it was noted by one HCP that one of their patients had chosen to do activity in their own way, although it is unclear if this was a conscious choice or the result of a misunderstanding about the aims of the Snacktivity approach.

I had one person in particular that was randomised to Snacktivity, delivered the Snacktivity intervention, and then when I come back … when she come back to see me, she had said, so what I’ve started doing is I started swimming three times a week for like an hour and a half, based on what you’ve said. I was kind of thinking, you’ve completely missed the point of Snacktivity, but great if that’s what you’re going to do and you’re happy with that, then that’s cool. (Male physiotherapist—WP3 HCP001)

A misunderstanding that was apparent related to patients expecting a more personalised approach from their HCP to help them become more physically active.

One or two people seemed to be expecting something else. One lady in particular, I think she was expecting a bespoke training programme to be delivered, you know where I’d tell her what she needed to do because she kept asking me. (Male podiatrist—WP2 HCP002)

### Perspectives about delivering the Snacktivity intervention in consultations

HCPs spoke at length about their experience delivering the Snacktivity intervention, in particular commentary centred on the elements impacting intervention delivery and highlighting specific barriers. Possible refinements to intervention delivery were also offered.

#### Delivering Snacktivity

HCPs were asked to follow a checklist of items to be mentioned or discussed with patients during the Snacktivity intervention. There appeared to be a preference for delivering the intervention at the end of the consultation. The checklist provided to the HCPs was considered useful in helping them deliver the intervention.

It was quite good to have the checklist to go through, it was quite straightforward to go through, I think it flowed quite well. (Female dentist—WP3 HCP007)

So maybe just advising people [future HCPs delivering the Snacktivity intervention] just to … to learn the check … go through the checklist before *(*Male physiotherapist*—WP3 HCP008*)

However, not everybody found it immediately helpful, and one HCP suggested that it could be reworked into a more user-friendly format, such as being in bullet points.

If it’s supposed to be used for clinicians as a prompt to deliver the intervention as they’re doing it, it is way too complex, there is way too much stuff on there and it’s very difficult to follow, it almost needs to be designed as if it was for a patient… (Male physiotherapist—WP3 HCP001)

Several HCPs altered the checklist to suit their working environment and personal style, and one used the checklist to develop their own ‘crib sheet’, which at least one other HCP used and identified as helpful in learning to deliver the intervention.

But you only had to do it sort of once and then you know you kind of like got the gist of it, which yeah, it … that really helped, that was really good (Female podiatrist—WP2 HCP004)

Because of the COVID-19 national lockdown period in England, some consultations were delivered by HCPs using the telephone, which prompted some reflection on the relative merits of telephone and in-person delivery for health behaviour change interventions such as Snacktivity. One HCP felt that telephone delivery allowed them to maintain control of the flow of conversation and thus deliver the intervention more quickly, although most HCPs preferred in-person delivery, citing benefits. These included reading body language cues better and an increase in trust from trial patients, leading to a possible enhanced commitment to the Snacktivity intervention. Another HCP felt that in-person delivery allowed for greater personalisation of delivery which is important for new healthcare interventions.

So I thought that was a bit more of a … it was much easier to do face to face, and I thought that obviously asking them about what they do day to day already, what their normal job is, what their commute is like, makes it easier to specifically choose a plan for them. (Female dentist—WP3 HCP009)

A few limitations were noted by the HCPs, with one being concerned that time constraints in practice might reduce the effectiveness and impact of the intervention.

So in order to squeeze it in, what I would worry about is making your message less effective and almost thinking, what’s the point of me even mentioning it, if you know what I mean? (Female dentist—WP3 HCP009)

Another HCP highlighted that there may not be the flexibility to allow the Snacktivity intervention to be delivered to all patients.

‘Because you know we’ve got twenty minutes or whatever it is, forty minutes for a new patient, and you wouldn’t be able to do the Snacktivity in that time realistically, you’d be running over’ (Male podiatrist-WP2HPC001)

#### Limitations, challenges and possible improvements

Some areas for improvement were noted by HCPs. The most frequently discussed barrier to the delivery of the intervention appeared to be the time required. Estimates of how much additional time the Snacktivity intervention added to a consultation ranged from 2 to 15 min.

So, it’s not five minutes. And if you’ve got a twenty-minute appointment of course, then there’s no way you’d be able to do the feet and deliver the intervention, unless you’ve got added time. (Male podiatrist—WP2 HCP001)

However, it was believed that the delivery time was likely to reduce with practice over time.

I just feel that we didn’t have enough practise doing it, obviously if it was a higher recruitment rate, it would have been much, much quicker to do that intervention. (Female dentist—WP3 HCP007)

Several HCPs indicated that intervention delivery was not burdensome.

Yeah, so it wasn’t too difficult to be fair (Male physiotherapist—WP3 HCP011)

This may have been facilitated by the steps some HCPs incorporated to ensure the smooth integration of the Snacktivity intervention within the consultation, with one composing their own approach to raising and discussing the topic of physical activity/Snacktivity.

…So I typed up my whole script and had little parts where I could extend off if I needed to, and I left little sort of pauses in between to check that the patient understood what I was saying. And I got them involved in between, just asking them a few little questions and things like that. (Male podiatrist—WP2 HCP003)

### Snacktivity intervention fidelity/audio recordings

Data from the HCPs (see table 1) who delivered the Snacktivity intervention were collected to assess intervention fidelity; community physiotherapists (n=3), hospital dentists (n=6), podiatrists (n=5) and healthcare assistants/nurses in general practices (n=13). Due to COVID-19, four consultations were delivered by researchers. A total of 46 Snacktivity interventions were delivered and recorded (study A: n=18; study B: n=28). On average, consultations lasted 10 min (range 2–28 min). In 6 (13.0%) consultations, HCPs discussed all 12 checklist items, and in 27 (58.7%), consultations 10–11 items were discussed. In 13 (28.3%), consultations 9–6 items were discussed. No HCPs discussed less than six items (table 3). The consultation items least discussed by HCPs were the importance of adhering to Snacktivity/physical activity over time and using strategies to facilitate this (43.5%; item 8) and checking that patients understood what the Snacktivity intervention involved at the end of the consultation (15.2%; item 11) (table 4).

**Table 3:** Snacktivity intervention checklist items discussed by HCPs in consultations

Number of checklist items mentioned (maximum score of 12)	Number of consultations which mentioned the intervention items (n=46, %)
12	6 (13.0)
11	15 (32.6)
10	12 (26.1)
9	5 (10.9)
8	5 (10.9)
7	2 (4.3)
6	1 (2.2)

HCPshealthcare professionals

**Table 4:** Frequency of delivery of the intervention checklist items by HCPs

Item number	Checklist items (in order presented)	Number of mentions Snacktivity interventions (n=46, %)
1	Mention the importance of physical activity for both physical and mental health. Mention the importance of physical activity to keep our muscles strong	44 (95.7)
2	Introduce the idea/concept of Snacktivity. Explain the specific advantages of Snacktivity	43 (93.5)
3	Emphasise the goal is to work towards achieving 30 min of moderate-vigorous intensity physical activity each day. This means they should raise their heart rate, for example, as if they were rushing for a bus	42 (91.3)
4	Suggest strategies that might help people to increase their Snacktivity/physical activity. (eg, planning when they might do Snacktivity or doing Snacktivity with somebody else	44 (95.7)
5	Mention how Snacktivity can help to reduce sitting time during the day.	42 (91.3)
6	Outline the purpose and importance of using the physical activity tracker (Fitbit) (provided after the consultation by research team).	46 (100)
7	Outline the purpose and importance of using the physical activity SnackApp (access provided after consultation by research team).	44 (95.7)
8	Mention the importance of trying to stick to Snacktivity/physical activity over time and using strategies to help them do this; the SnackApp will have lots of ideas.	20 (43.5)
9	Mention the importance of action planning (really encourage the patient to think about where and when they will do their Snacktivity/physical activity).	34 (73.9)
10	Check the participant has set an initial goal and highlight how the SnackApp can help them do this (point them towards the schedule feature on the SnackApp).	32 (69.6)
11	Check the patient understands what the Snacktivity intervention involves.	7 (15.2)
12	Check the participant knows where to find any further information if they have any questions/problems.	38 (82.6)

## Discussion

Following the ambitions of the MECC initiative, this study aimed to explore the ‘experiences of a diverse group of HCPs in delivering the Snacktivity intervention within routine health consultations. HCPs understood the aims and purpose of Snacktivity and believed the approach could be beneficial to a wide range of populations. There was a view that Snacktivity could help to develop more positive views about physical activity in patients, which can be a difficult health behaviour to change and sustain. HCPs commented that their patients were generally enthusiastic about the Snacktivity concept and were open to conversations about it in consultations. While HCPs generally felt positive about including conversations about Snacktivity in consultations, several operational and clinically orientated barriers to doing so were raised. The audio recordings demonstrated that HCPs delivered the Snacktivity intervention with high fidelity, although they were not always able to do so in the intended timeframe of 5–7 min. This finding is nevertheless encouraging given that several of the HCPs in the study would not usually promote physical activity with their patients (ie, dentists). Our findings contribute to evidence for MECC-based programmes to support preventive health policies, which have not been well developed to date. This study also highlights the potential that MECC approaches may have for promoting physical activity at scale or within whole healthcare systems.

### Usefulness of MECC to promote Snacktivity

Our finding that HCPs considered MECC as potentially useful in promoting Snacktivity to their patients is consistent with studies that have examined other health behaviours. For example, Chisholm *et al* explored public health practitioners’ views of implementing the MECC initiative and reported that they felt it was potentially a valuable approach for improving public health.^25^ It was encouraging to see that HCPs felt the training they received was appropriate and well delivered, not least because training is likely to be integral to engagement from HCPs in health behaviour interventions. However, consideration must also be given to the paradox that HCPs can find conversations about health behaviour change difficult and believe them to be ineffective,^12 13^ yet we know that patients welcome the opportunity to have these types of conversations with healthcare providers, as a way of supporting them to improve their health.^2^

### HCPs confidence/competence to deliver health behaviour change interventions

HCPs were trained to deliver the Snacktivity intervention, where the focus was on learning the principles and theoretical basis of Snacktivity, how to integrate health behaviour change conversations within consultations and on explaining the supporting Snacktivity intervention technology. Some HCPs expressed greater confidence in delivering the intervention than others, and this may be associated with the purpose of the consultation and/or the professional background of individual HCPs. For example, it is more natural for podiatrists and physiotherapists to discuss physical activity and movement with their patients than is the case for dentists (also see the ‘Discussion’ section). This raises an interesting question about health services addressing the additional training needs of HCPs, many of whom may not consider themselves sufficiently skilled to deliver health behaviour change interventions within the consultation time available. Moreover, while the discussions about Snacktivity were designed as a brief intervention to take HCPs between 5 and 7 min to deliver in consultations, the audio recordings indicated that in fact the average was 10 min. This highlights that it is not always possible for HCPs, to deliver health behaviour interventions in a few minutes, or within a standard consultation period. That said, over time and with more practice, it is possible that HCPs can become more efficient at explaining and delivering these types of preventive health interventions within consultations.

### Professional role and preventive medicine

HCPs have contact with a large proportion of the population and have a key role to play in promoting physical activity to their patients. Our findings are consistent with a systematic review that addressed the delivery of behaviour change interventions by HCPs, which found that many view discussions about health behaviours outside of their expertise and scope of their work.^26^ For example, in this study, dentists raised that initially conversations about Snacktivity felt out of place as such topics are not consistent with the professional role of a dentist. Moreover, patients who attend an appointment at a dental clinic because of tooth pain are not expecting their dentist to begin a conversation about physical activity/Snacktivity with the consultation. This highlights a question about the viability of MECC approaches, which has the principal aim of encouraging all HCPs, regardless of professional background or the healthcare context, to deliver behaviour change messages in consultations. We know from other studies that HCPs can be reticent about raising topics related to preventive medicine in consultations for several other reasons, a common one being, fear of not knowing how to best support patients.^27^ Other qualitative research in the UK has reported that HCPs were positive about the value of MECC idea and health behaviour change based interventions, but they had concerns about their ability to deliver such interventions, and that the work environment within health services limits the opportunity for them to engage with this approach.^12^

While there may be strengths to the Snacktivity approach to promoting physical activity, Snacktivity may not always be consistent with the therapeutic requirements of a treatment being offered to patients. In the case of physiotherapists in the study, there may be a need to prescribe specific sustained exercise(s), rather than repeated small activity snack bouts, resulting in conflicting information being given to patients.

### Time needed to treat

MECC-based initiatives are reliant on HCPs being willing to deliver health behaviour change messages within consultations. While HCPs did not find the intervention burdensome to deliver, consistent with other studies, some HCPs raised the issue of having sufficient ‘time to treat’ in the event they were required to discuss preventive health with all patients, not just the few they saw as part of this study. Like other studies, limited time was viewed as a tangible barrier to implementation of preventive health programmes by HCPs.^26^ This highlights that the time allocated for health consultations may need to be addressed and increased if MECC is to be fully implemented in a whole systems healthcare approach.^28^ Specifically, a tension may exist between the need to deliver core acute healthcare services and the ambition of health services to also reduce diseases via preventive health programmes. The concerns of HCPs about the additional time required to deliver MECC interventions speaks to the need for system-level changes in health services to allow prevention initiatives to be appropriately implemented by HCPs.

### Delivery of the Snacktivity intervention within consultations

Audio recordings of consultations can provide an objective means of assessing how well HCPs are able to deliver health behaviour interventions within their practice. This type of data can also highlight where further training may be needed, particularly when delivering a new intervention or service, or whether the training content needs to be further developed It was encouraging to see good levels of intervention fidelity by HCPs. It is acknowledged, however, that HCPs were only required to deliver a small number of consultations each and fidelity may be lower if they needed to do the same with every patient given the time pressures that they face.

### Strengths and limitations

The findings of the study should be interpreted in light of its strengths and limitations. This is the first study to explore HCPs’ experiences of promoting the Snacktivity approach within routine consultations. HCPs from a wide range of clinical backgrounds were recruited. A large number of audio recordings from consultations where HCPs delivered the Snacktivity intervention were obtained to allow for the assessment of intervention fidelity. Studies such as this one are important in helping to inform the design of future research on this topic and further work to assess the effectiveness and cost-effectiveness of the Snacktivity intervention in a variety of health and public health settings is ongoing (https://www.isrctn.com/ISRCTN12390945). The study also has some limitations. It is possible that in a research context where the interactions of HCPs with patients were being recorded, they may have performed better than would be the case within their everyday clinical practice. Convenient samples of HCPs and patients/participants were recruited and this may mean that the findings are specific to the HCPs who were willing to take part. A modest number of HCPs were interviewed, and future research should consider including a larger sample to capture a broader range of views.

## Conclusion

Strategies to support the public to change their health behaviours have the potential to reduce the burden of disease in the population. However, the success of this approach is dependent on HCPs being willing and able to deliver these consultations and patients engaging with the advice. While HCPs initial views were supportive of delivering a relatively simple, structured intervention to help patients become physically active through Snacktivity, some barriers to implementation were identified, including time and confidence to deliver the intervention. This study has highlighted the importance of developing health professionals’ skills in facilitating behaviour change, to making consultations that they deliver contribute towards reducing diseases in the population.

## supplementary material

10.1136/bmjopen-2024-085233 online supplemental file 1

10.1136/bmjopen-2024-085233 online supplemental file 2

10.1136/bmjopen-2024-085233 online supplemental file 3

## Data Availability

No data are available.
