# Supplementary material for: Making Every Contact Count: health professionals’ experiences of integrating conversations about Snacktivity to promote physical activity within routine consultations – a qualitative study
Source: BMJ Open. 2024 Oct 22;14(10):e085233. doi: 10.1136/bmjopen-2024-085233 (PMC11499785; doi:10.1136/bmjopen-2024-085233)
Supplement: online supplemental file 1 [file bmjopen-14-10-s001.pdf]

Appendix 1.

HCP interview schedules

NON PARCTICPANT FACING : RESEARCHER USE ONLY

Participants semi-structured interviews (Snacktivity intervention group)

These questions are provided as overarching objectives, but questions and prompts may be

developed as the discussion develops to incorporate any important themes that emerge from the conversation. Not all questions will be presented to all participants, these questions are prompts.

I'd like to talk with you today about your thought of delivering the Snacktivity intervention as part of

your consultations with patients. We would also like to hear about your experiences of attending the training and being a part of this study.

Warm up

- How long have you been a practice nurse/podiatrist/ physiotherapist/ OT/dietician?
- Prior to delivering Snacktivity- what information (if any) did you provide patients about physical activity?
- [for those not providing this] – why is this the case?

Reasons for participating in the trial

I would like to move on now and talk a bit about why you decided to take part in the Snacktivity study

- I would be interested to know what made you decide to take part?
- Can you tell me about what it has been like for you being involved in the Snacktivity study and discussing Snacktivity in your consultations?

- What were you hoping to get out of being a part of the Snacktivity study?

General views about Snacktivity

- What are your thoughts on the idea of Snacktivity?
- Does the Snacktivity approach make it easier for you bring up physical activity in your consultations?

Training in Snacktivity

Now I'd like to talk about the training you received to deliver the Snacktivity intervention to patients in your consultations

- Could you describe the training you received for this trial?
- What did you think of the training? What did you like and dislike?
- What do you think we could have added or changed about the training session to make it more useful for you?
- What did you think of the videos and the training consultations we showed you?
- After the training, how prepared did you feel to deliver the Snacktivity intervention?

Appointments

I would now like to switch topics and talk about what happened during your consultations with patients

when you were taking about Snacktivity?

- How easy was it to identify patients who were taking part in the trial?
- Could you walk me through what would happen during the consultation when you talked about Snacktivity with patients?

 Can you tell me how you felt knowing that you would be talking about Snacktivity to some of your patients? How comfortable did you feel? How confident did you feel?

 Could you tell me a bit about what sort of things you'd say to patients about Snacktivity?

 How comfortable do you think the patients were with you talking about Snacktivity with them?

 How much more time did consultations take when you had to discuss Snacktivity?

 Can you tell me what you think it would be like if you had to routinely discuss Snacktivity with all your patients?

 Did patients have many questions about Snacktivity?

 Did patients have any questions about using a SnackApp or the SnackApp watch? Is there anything we could do to make this process smother for you?

SnackApp and Snacktivity watch

Now I want to ask you about the SnackApp and tracker

 Were you able to have a look at the SnackApp (and device)? What did you think of it?

Additional questions

 Can you tell me what sort of things we could have done differently to make things easier for you?

 Can you tell me what would you think we should have done to make being involved in the trial better for you?

 Is there anything else you'd like to tell me?
