# Supplementary material for: Making Every Contact Count: health professionals’ experiences of integrating conversations about Snacktivity to promote physical activity within routine consultations – a qualitative study
Source: BMJ Open. 2024 Oct 22;14(10):e085233. doi: 10.1136/bmjopen-2024-085233 (PMC11499785; doi:10.1136/bmjopen-2024-085233)
Supplement: online supplemental file 2 [file bmjopen-14-10-s002.pdf]

Appendix.2

Snacktivity™ intervention fidelity checklist for health care professionals

DON'T FORGET TO TURN ON YOUR DICTAPHONE (with patient consent)

Participant trial number: __ __ __ __ __ __   Recording number: __________________

Date: __ __ / __ __ / __ __ __ __

Is the consultation being held: Online/via telephone ☐ *or* face to face ☐

**Checklist item**	**Yes**
Mention the importance of physical activity for both physical and mental health. Mention the importance of physical activity to keep our muscles strong.	
Introduce the idea and concept of Snacktivity. Explain the specific advantages of Snacktivity.	
Emphasise the goal for participants is to work towards achieving 30 minutes of Moderate-to-Vigorous Physical Activity (MVPA) per day. This means they should raise their heart rate, for example as if they were rushing for a bus.	
Suggest strategies that might help people to increase their Snacktivity/physical activity. For example, planning when they might do Snacktivity or doing their Snacktivity with somebody else	
Mention how Snacktivity can help to reduce sitting time during the day.	
Outline the purpose and importance of using the physical activity tracker (to be provided by the research team after this appointment).	
Outline the purpose and importance of using the physical activity SnackApp (access to this app is provided after this consultation).	
Mention the importance of trying to stick to Snacktivity/physical activity over time and using strategies to help them do this; the SnackApp will have lots of ideas.	
Mention the importance of action planning (really encourage the patient to think about where and when they will do their Snacktivity/physical activity).	
Check the participant has set an initial goal and highlight how the SnackApp can help them do this (point them towards the schedule feature on the SnackApp).	
Check the patient understands what the Snacktivity™ intervention involves.	
Check the participant knows where to find any further information if they have any questions/problems.	

Name of healthcare professional: ____

Occupation/role: ________________________________

Signature: _____________________________________
