# Supplementary material for: Making Every Contact Count: health professionals’ experiences of integrating conversations about Snacktivity to promote physical activity within routine consultations – a qualitative study
Source: BMJ Open. 2024 Oct 22;14(10):e085233. doi: 10.1136/bmjopen-2024-085233 (PMC11499785; doi:10.1136/bmjopen-2024-085233)
Supplement: online supplemental file 3 [file bmjopen-14-10-s003.pdf]

Appendix 3

The lived experience of the non-delivery elements of being in a clinical trial

Substantial discourse relating to the non-delivery elements of HCPs Snacktivity™ experience were apparent, these are the elements which were unrelated to the frontline delivery of Snacktivity and thus covered topics such as the HCP's experience of their training and the resources of that training and their engagement with the trial elements of the study, such as recruiting participants.

<u>Engagement with the trial</u>

HCPs were generally positive about participating in the trial and the Snacktivity™ approach to promoting physical activity.

> "I've always had an interest in exercise and keeping healthy. And I really see an enormous amount of value in this approach because I think by encouraging people into these short activity Snacks it will motivate them and if they can see the progress that they're making without making huge changes to their lifestyle, then you know it will drive them forward to become more and more active, and obviously the knock-on effects for their general health." (WP2 HCP02)

It was apparent that the decision to participate in the trial had taken in many factors, with HCPs often mentioning multiple reasons. Amongst these, being directly asked by a colleague, especially one in authority, was mentioned by several of the HCPs (WP3 HCP 001, WP3 HCP 007, WP3 HCP 011).

Personal engagement with the value of physical activity was also important to some HCPs.

> "I actually thought that the subject itself was of an interest to me too." (WP3 HCP 010)

> "I guess I really do believe that physical activity is vital, I think it's something that especially the healthcare system, being overwhelmed, I think physical activity can

> help a large proportion of people coming in. So I think as a preventative kind of measure, physical activity's really important …" (WP3 HCP 008)

A further factor was the career's demands to be engaged with research to some degree (WP3 HCP-001, WP3 HCP 008, WP3 HCP 009, WP3 HCP-010).

> "So my main motivation was because within my role it is to facilitate research within the service and also the wider Trust, not necessarily because it was around physical activity and I thought there would be a benefit to that." (WP3 HCP 001)

> "This is more so for career development aspect as at the time I was applying for an academic post and I wanted to expand my research experience." (WP3 HCP 010)

And/or

> "I hadn't been involved in any research projects before, so I wanted to have an experience of that, and I thought it was quite an interesting idea for a trial as well." (WP3 HCP 009)

Participating in research did bring benefits to the HCPs, predominant amongst these were indicators of a deeper understanding of the research process (WP3 HPC-07 and WP3 HPC-011). and there was a sense that the HCPs were enthused by involvement with the research.

> "No, I think … I think it was great, yeah, thank you for involving me in this study because it was … it was good, I enjoyed being part of it." (WP3 HCP – 010)

An unexpected benefit from participation for one of the HCPs was introducing the Snacktivity™ approach to a family member. (WP2 HCP02)

> "So, he tried to do some exercise himself and couldn't … literally couldn't get … walk around the block. So, to tell my brother, xxxx, you need to do thirty minutes of exercise, he would be lying flat on his back well before that. So, you know I tried this approach with him, saying just do a bit, get up, set a timer on your phone like every hour and get up and walk up and down the stairs or do

some squats or you know swing your arms around! Do little bits." (WP2 HCP02)

However, there were expressions of exasperation with the number of emails that came with being part of the trial.

"I know a couple of my colleagues got a little bit overwhelmed with the amount of e-mails and communication that they were being copied into." (WP2 HCP 02)

Reflections on the training experience to deliver the Snacktivity™ intervention

HCPs identified that they felt prepared by the training they received (WP2 HCP02, WP2-HCP03, WP2-HCP04).

"… she was very clear about everything, what Snacktivity™ was and what was expected from us, how we were going to go about it." (WP2 HCP03)

Whilst HCPs in study B received their Snacktivity™ training online due to the COVID-19 restrictions, this appeared to have had no effect on its perceived quality with several HCPs expressing that they felt prepared at the end of the training (WP3-HCP007, WP3-HCP008, WP3-HCP010). Further, there is evidence that HPCs initiated in-house training once sufficient experience had been established in the setting.

"I had quite good training from the colleague I mentioned as well, who had done a few of them." (WP3 HCP 009)

Some of the HCPs (WP2 01 & 02) identified the training video as particularly useful in helping them to learn how to deliver the Snacktivity™ intervention within their health consultations.

"That was because I could go back to it and you know look at it in my own time, see how the … because that was I suppose the critical thing for me … I mean I knew roughly about the background and everything, but it was you know more about how do you deliver it and the sorts of things you would say, because never

> having done it, you know, you don’t know how much depth to go into or you know what you skim over or whatever, how you word things. So I found the video very useful, I looked at that about a couple of times, two or three times.” (WP2 HCP01)

And/or

> “But I found the video was very clear, it was quite short, which was good as well … and when I sent it out to my team, they also really liked … it was quite easy for them to train them using that video as well” (WP3 HCP 007)

The video was however perceived by some as being representative of how an idealised patient encounter might go and was not a true representation of a real-world encounter.

> “Yeah, that was really useful to go back and look at, to … for prompts and reminders, so we used that when we were doing our little one to ones before seeing patients, we would use that video. You’ve got to use real patients though in the video! Because it’s just … someone going, yeah, that’s great, yeah, that’s fine, that is not how patients work!” (WP3 HCP 001)

For training purposes, the video was initially shown in segments to highlight elements of the intervention, there was however a desire for it to be presented in full (WP2 HCP02).

> “The video itself was useful, but I would have liked to see like a full delivery, I think it was in bits really wasn’t it?” (WP3 HCP 011)

Another HCP would have liked the training video to cover elements of what happens to patients prior to, and after receiving their Snacktivity™ intervention (WP2 HCP01) to provide further context.

In comparison to the video, the written materials of the study drew relatively little commentary, with only two evaluative examples arising, one of which highlighted the functionality of this material.

"So we had the … there was … we had lots of documents that we could go back and refer to, which we did, they were useful." (WP3 HCP 001)

However, the other example demonstrated a sense of overwhelm with the study materials when examined prior to training.

"Yeah, and I thought, ooh I don't know whether I can do this, and then I started questioning my own you know competency, you know, I thought oh no I don't think I can do this, I felt sort of frightened, it made me feel fearful, and that didn't make me feel very good really. But you know long, short, when you do deliver it, it's quite simple, it's … you know the process is, in reality, in real time, it's very simple, but all the paperwork that went with it beforehand and stuff." (WP2 HCP04)

One HCP noted that the training left them with a lack of knowledge about the technology to support the intervention and trial.

"I mean I'm probably going off at a bit of a tangent, but I wasn't really clear about the fit app and all the apps and the watches and … I didn't realise that they had one just to get the background check and then they'd have another one …" (WP2 HPC01)

Several HCPs made suggestions for improving the training module including role-playing the intervention (WP3 HCP001, WP3 HCP008, WP3 HCP010 WP2 HCP01 & WP2 HCP04)

"No, the only thing I'd say is I'd probably like … given the opportunity again, it would probably be good practice going through interventions" (WP3 HCP 008)

Other suggested improvements included; being participants in Snacktivity™ (WP2 HCP04), conducting the training closer to the time of delivery (WP2 HCP01 &WP2 HCP03), or

providing refresher training (WP3 HCP 001)

> "Yeah, a refresher or just a catch-up at some point, or … I know it was recorded, but having some feedback on that, saying you did a really good job with this, this and this, it would be good if you did a bit more of this or you missed these points, so actually having some of that feedback would have been useful." (WP3 HCP 001)

Being given the opportunity to experience the intervention technology was also suggested (WP2 HCP01 & WP2 HCP04),

> "I think it's a matter of just encouraging like us, the clinicians that you know it's easy, it's fine, don't worry, you know, it's straightforward once … you just have to have a go and do it. And maybe do some sort of role play perhaps on the day of that training, it might have you know just give a bit more of a flavour of how it is you know?" (WP2 HCP04)
>
> And/or
>
> "…if you're a keen healthcare professional, you might potentially go in, have a bit of a play with it yourself, so you feel that you know more about what you're talking about when you talk to somebody! " (WP2 HCP05)
