# Supplementary material for: Making Every Contact Count: health professionals’ experiences of integrating conversations about Snacktivity to promote physical activity within routine consultations – a qualitative study
Source: BMJ Open. 2024 Oct 22;14(10):e085233. doi: 10.1136/bmjopen-2024-085233 (PMC11499785; doi:10.1136/bmjopen-2024-085233)
Supplement: Supplementary file 4 [file bmjopen-2024-085233.reviewer_comments.pdf]

PEER REVIEW HISTORY

BMJ Open publishes all reviews undertaken for accepted manuscripts. Reviewers are asked to complete a checklist review form and are provided with free text boxes to elaborate on their assessment. These free text comments are reproduced below.

ARTICLE DETAILS

**TITLE (PROVISIONAL)**	Making Every Contact Count: Health professionals' experiences of integrating conversations about Snacktivity™ to promote physical activity within routine consultations: A qualitative study
**AUTHORS**	Krouwel, Matthew; Greenfield, Sheila; Sanders, James; Gokal, Kajal; Chalkley, Anna; Griffin, Ryan A; Parretti, Helen; Jolly, Kate; Skrybant, Magdalena; Biddle, Stuart; Greaves, Colin; Esliger, Dale; Sherar, Lauren; Edwardson, Charlotte; Yates, Thomas; Maddison, R; Frew, Emma; Mutrie, Nanette; Ives, Natalie; Tearne, Sarah; Daley, Amanda

VERSION 1 - REVIEW

**REVIEWER NAME**	Christopoulos, Konstantinos
**REVIEWER AFFILIATION**	University of Cyprus
**REVIEWER CONFLICT OF INTEREST**	No competing interests
**DATE REVIEW RETURNED**	01-Mar-2024

**GENERAL COMMENTS**	This study examines (qualitatively) the difficulties in implementing a physical activity (PA) intervention, Snacktivity, in various heart care settings. The importance of these interventions is undisputed but there are many issues with their delivery by health care professionals (HCP), the way NHS operates, as well as inconclusive evidence regarding their effectiveness.<br><br>There are some, perhaps unavoidable, methodological issues. First, the convenience sampling of HCP does not allow for generalisation of the results and patients were also self-selected in study A so this does not provide an accurate picture of how this intervention would go in real settings. Second, a recorded performance/intervention delivery may be different from when unrecorded. This can go both ways but usually HCPs will do better when observed. This is akin to the Hawthorne effect.<br><br>The selection of diverse HCP offers a bigger picture but also limits the numbers of each practice. I am skeptical about the inclusion of dentists. Indeed, an appointment to the dentist is the last place you expect to hear PA advice. On the other hand, the patient cannot talk when receiving treatment so the dentist can initiate whatever "conversation" he/she likes. But this one-way conversation is probably not ideal and may not be enough. It not clear when during treatment these interventions were delivered but dental appointments can be very stressful for some individuals and I doubt whether they would pay attention to PA talk before, after, or during their treatment.<br><br>I believe that due to heterogeneity in HCP, a tailored approach should be developed if the intervention is to be implemented from

	such diverse practitioners. For example, PTs would have to clarify that this intervention is not the rehabilitation for the injury they are facing but it may help them in the long run.<br><br>It is also unclear how this intervention can be delivered in a typical 10—15’ NHS appointment in primary care. A systematic change is extremely unlikely to happen since the main goal of the system is drug prescription (necessary or not). An option would be for GPs to initiate the intervention and then give a brochure and/or refer interested patients to another professional. This will take only a couple of minutes and can be cost-effective.<br><br>On the other hand there is evidence that simple verbal interventions for PA in primary care do not really work (Hillsdon et al., 2002; van Sluijs et al. 2004) and results are mixed for more complex interventions (Pavey et al., 2011; Arrow et al., 2012). Supervised PA programmes, on the other hand, appear to have some success (Dinan et al., 2006). This is not a simple intervention of course, but only certain HCPs will have the time (and willingness) to deliver it. So it cannot make every contact count as intended.<br><br>It is important to advance to a quantitative study so that HCP profiles that struggle in delivery can be identified. Ideally, with patient follow-up the relative effectiveness of interventions can be studied.<br><br>It would also be interesting to study the effect of these interventions and the difficulties in treatment delivery on hard-to-reach populations which may be some of those most in need of PA.<br><br>All in all, the study is well written and conducted but I expect some additional discussion on the limitations and plans for future research that will provide more concrete evidence on the matter.<br><br>Minor comments<br><br>I agree that younger generations of HCP are more likely to engage in this topic but please provide a reference p.6 lines 9—11.<br><br>p.22 line 17 It is better to write 10—11 or 10 to 11 items (10/11 resembles a proportion)<br><br><br>References<br><br>Hillsdon M, Thorogood M, White I, Foster C. Advising people to take more exercise is ineffective: a randomized controlled trial of physical activity promotion in primary care. Int J Epidemiol. 2002;31(4):808-815. doi:10.1093/ije/31.4.808<br><br>van Sluijs, E.M., van Poppel, M.N. and van Mechelen, W., 2004. Stage-based lifestyle interventions in primary care: are they effective?. American journal of preventive medicine, 26(4), pp.330-343.<br><br>Pavey TG, Taylor AH, Fox KR, et al. Effect of exercise referral schemes in primary care on physical activity and improving health outcomes: systematic review and meta-analysis. BMJ. 2011;343:d6462. Published 2011 Nov 4. doi:10.1136/bmj.d6462

	Dinan S, Lenihan P, Tenn T, Iliffe S. Is the promotion of physical activity in vulnerable older people feasible and effective in general practice?. Br J Gen Pract. 2006;56(531):791-793.<br><br>Orrow G, Kinmonth A, Sanderson S, Sutton S. Effectiveness of physical activity promotion based in primary care: systematic review and meta-analysis of randomised controlled trials BMJ 2012; 344 :e1389 doi:10.1136/bmj.e1389

**REVIEWER NAME**	Fowles, Jonathon
**REVIEWER AFFILIATION**	Acadia University, Kinesiology
**REVIEWER CONFLICT OF INTEREST**	I do not have any competing interests with anyone in the author group for that paper.
**DATE REVIEW RETURNED**	15-Mar-2024

**GENERAL COMMENTS**	Overall comments to the authors<br>This evaluation is a small component of what looks like a very large intervention to implement Snacktivity as part of the MECC framework across healthcare. Given the timing of the intervention, and by the authors descriptions in the text, the COVID pandemic likely had a significant effect on health care provider engagement in the study and likely the ability to collect data on patient interactions. Given the MAJOR burden shouldered by Health Care Providers (HCPs) during this timeframe, it is with this lens that primary criticisms to the robustness of the evaluation are tempered. Having said this, the major limitation of the study is the small number of HCPs evaluated and the brief exposure/experience they had with the intervention – most HCPs only implemented it with 2 patients and the most was 4, which is insufficient to make conclusions about ‘integrating’ the intervention into ‘routine’ consultations. By the authors own admission , the target intervention delivery time of 5-7 min was not achieved (10min), likely due to the HCPs’ lack of comfort delivering the intervention (i.e. most working from a script, and most acknowledging in extra comments in the end that more time to ‘work through’ or practice the intervention would have been beneficial).<br>For these reasons, it is important for the authors to contextualize these limitations to the study, in the title, abstract and conclusions; there are also some terms that could be changed to clarify the parameters of the study. There is a lack of research on implementation of these system wide changes to practice, so the study has merit, especially the issue of implementing physical activity behaviour change into health fields that might not normally be doing lifestyle-related counselling (e.g., dentistry). The authors seemed to be hiding who the ‘health providers’ were until the methods. This can actually be reframed as a strength of the study – to really evaluate the reach of the Snacktivity within the broad framework of MECC across ALL healthcare. See suggestions below.<br>Title. Recommend editing the title to more closely reflect the context of the study. Note: as the term ‘consultations’ implies that patients are coming in to discuss medical issues, whereas they may have been coming in to get a dental filling, and then had a conversation about physical activity.<br>Health professionals’ initial experiences integrating conversations about Snacktivity™ to promote physical activity within typical medical appointments<br>Abstract

	Study question: 'explored' experiences of HCPs delivering. Yes, this is accurate given that the interviews are open ended questions to explore their experience, but, there is a vast amount of literature on the challenges that HCPs face Based on previous research couldn't' there be a preliminary question/hypothesis?<br>2nd: HCP ability/fidelity to deliver Snacktivity<br>Results: 'conceptualisation' of Snacktivity – is this term apparently clear? This reviewer did not completely understand the intent of this term until reading through the whole paper. This is health providers' 'conceptualisation'.<br>'Consulation'. Clarify as 'health professional-patient consultation' as it was unclear initially. This might be a language-term as medical appointments in N America are not normally referred to as 'consultations'.<br>'practical barriers to implementation' be forthwrite with this. Although time training/education and confidence were barriers implementation.<br>Conclusions: this sentence did not make sense until reading the whole paper. Why would developing skills in health behaviour change affect practical barriers to implementation if the fidelity was high? (i.e. they had the skill to deliver the intervention as prescribed). Its because their lack of experience with it meant it took more time than intended. So consider restating:<br>"Health professionals were proficient and supportive of delivering the Snacktivity intervention within medical appointments. The primary barrier to implementation was the time to deliver it, however, gaining greater experience with the intervention and improving behaviour change counseling skills may reduce that barrier."<br>Strengths and limitations –<br>Bullet 2 – the merits of a diverse set of healthcare professionals…… in typical consultations.<br>Bullet 3 – in a wide range of health settings<br>Bullet 4 - 'modest' (small). consultations 'Provider-Patient' consultations?<br><br>INTRO<br>Good set up of the paper, although all references are UK focused. There is a large body of research from the Exercise Is Medicine initiative out of the N.America that has delineated the barriers to implementation of physical activity counselling in medical practice. Barriers such as time, education/training, confidence have all be previously shown to influence the implementation of such initiatives, so it is somewhat confusing as to why there is a need for an 'open exploration' of this topic. What did the researchers think they would find that is outside of the scope of the previous research on the topic?<br>METHODS<br>Pg 8 line 7. HCPs across a wide diversity of health professions were recruited….. to test feasibility delivering the intervention with all areas of health services, including areas that may not normally be involved in lifestyle related behaviour change counseling such as ….((i.e. lean into this as initially it is odd that podiatry and dentistry were included in a PA intervention, but later in discussion it makes sense. So bring this intent forward).<br>The 'intervention' study here is not entirely clear. It is explained later (page 8 lines 3-16) Consider moving this section up front in the methods, because studies A and B evaluated here, are subsets of the Snacktivity intervention project. It<br>RESULTS:

	Only 2 pod delivered more than 3 consults. 5 of them 2 consults, 1-1. This is not sufficient experience with the module to make informed comments on it. References to this feedback is then 'initial experiences' not a fully informed regular practice using this intervention over several months with hundreds of patients as what would qualify 'implementation in routine care'. Authors need to change language throughout to acknowledge this.<br>Quotes – somewhat representative; however, female dentist 007 and 010 compose 9 out of 23 quotes.<br>5 podiatrists, 3 dentists 3 physios. Is this representative of 'health care delivery' the way the MECC was intended? AS mentioned previously – it is a diverse group that would not normally be expected to deliver physical activity advice, so the authors should lean into this as a strength of testing the MECC framework.<br>Pg 22 – Assessing fidelity. In my opinion, following a 'script/checklist' does not display confidence/competence in delivering a counseling intervention. It typifies a lack of comfort with it, but may be required to fulfill the intervention requirements.<br>Discussion<br>Pg 25 line 5 - delivering the intervention 'well' would be within the allotted time (i.e. 5 min). Delivered the intervention with 'high fidelity', although were not able to do it in the intended timeframe, possibly due to a lack of experience with behaviour change counseling and/or the intervention itself.<br>The rest of the discussion clearly outlines the paradoxical challenge of a 'whole of health care' approach to behaviour change counseling (i.e. MECC), so this intent to 'explore' this challenge, needs to be clear up front and throughout the paper. See comments above.<br>The rest of the discussion articulates the challenges and considerations relating to this effectively.<br>Conclusions. Pg 28 l12 'some barriers' don't hide what these are. Be clear and forthright with it. Time and comfort/confidence.<br><br>References<br>Note ref 4 is to be cited as:<br>Health Service Executive (2016). Making Every Contact Count: A Health Behaviour Change Framework and Implementation Plan for Health Professionals in the Irish Health Service. Health Service Executive. ISBN No 978-1-78602-038-3

VERSION 1 – AUTHOR RESPONSE

Reviewer: 1

This study examines (qualitatively) the difficulties in implementing a physical activity (PA) intervention, Snacktivity, in various heart care settings. The importance of these interventions is undisputed but there are many issues with their delivery by health care professionals (HCP), the way NHS operates, as well as inconclusive evidence regarding their effectiveness. There are some, perhaps unavoidable, methodological issues. First, the convenience sampling of HCP does not allow for generalisation of the results and patients were also self-selected in study A so this does not provide an accurate picture of how this intervention would go in real settings. Second, a recorded performance/intervention delivery may be different from when unrecorded. This can go both ways but usually HCPs will do better when observed. This is akin to the Hawthorne effect.

Response: The reviewer has raised some interesting points that we would certainly agree with. We apologise but due to an editing issue the strengths and limitations section was omitted from the original manuscript but we have included this now. Please see page 27. Regarding effectiveness,

studies such as this one are important in helping to inform the design of a future effectiveness trial and we have added a comment about this on page 27. Regarding generalisation, this is not the purpose of qualitative research, so we have declined to include this comment in the manuscript.

The selection of diverse HCP offers a bigger picture but also limits the numbers of each practice. I am skeptical about the inclusion of dentists. Indeed, an appointment to the dentist is the last place you expect to hear PA advice. On the other hand, the patient cannot talk when receiving treatment so the dentist can initiate whatever "conversation" he/she likes. But this one-way conversation is probably not ideal and may not be enough. It not clear when during treatment these interventions were delivered but dental appointments can be very stressful for some individuals and I doubt whether they would pay attention to PA talk before, after, or during their treatment.

Response: The Snacktivity™ intervention has been designed such that any health care professional should be able to deliver it. The dentists provided the intervention before or after patients received their dental treatment. Dentists and dental teams are involved in delivering health behaviour change advice/support to their patients in many countries. Some example reference for the reviewer to refer to regarding this are listed below:

Doughty, J., M. Gallier, S., Paisi, M., Witton, R. & J. Daley, A. Opportunistic health screening for cardiovascular and diabetes risk factors in primary care dental practices: experiences from a service evaluation and a call to action. British Dental Journal 2023 235:9 235, 727–733 (2023).

Large, J. F., O'Keefe, E., Valentine, C. & Roebuck, E. M. Weight screening in paediatric dentistry: What do families and staff think? International Journal of Paediatric Dentistry 32, 64–66 (2022).

I believe that due to heterogeneity in HCP, a tailored approach should be developed if the intervention is to be implemented from such diverse practitioners. For example, PTs would have to clarify that this intervention is not the rehabilitation for the injury they are facing but it may help them in the long run. A systematic change is extremely unlikely to happen since the main goal of the system is drug prescription (necessary or not). An option would be for GPs to initiate the intervention and then give a brochure and/or refer interested patients to another professional. This will take only a couple of minutes and can be cost-effective.

Response: There are a number of models and approaches where all health professionals (not just GPs) might deliver health behaviour change interventions to the public. Our approach followed the MECC approach, as many other studies have done, but it is not the only approach that can be taken. Governments around the world are paying much more attention to the role of preventative health care and we do not agree with the comments that systematic change is unlikely or cannot happen. It is more likely to happen when there is evidence it can be effective, which is the purpose of this programme.

It is also unclear how this intervention can be delivered in a typical 10—15' NHS appointment in primary care. A systematic change is extremely unlikely to happen since the main goal of the system is drug prescription (necessary or not). An option would be for GPs to initiate the intervention and then give a brochure and/or refer interested patients to another professional. This will take only a couple of minutes and can be cost-effective.

Response: The intervention is designed to be delivered across health care settings, not just primary care. In this study the intervention was delivered in primary care, community care and secondary care. We have commented about systematic change in the response above. If the Snacktivity™ intervention were to be included within routine health care then a time allowance for this to happen

would be needed, or as the reviewer points out, it may be that other modes of delivered are considered. Based on the reviewer comment below we believe the reviewer may have missed all the components, which is much more than a consultation and involves an important technology component. See page 8 for a detailed description of the intervention.

On the other hand there is evidence that simple verbal interventions for PA in primary care do not really work (Hillsdon et al., 2002; van Sluijs et al. 2004) and results are mixed for more complex interventions (Pavey et al., 2011; Arrow et al., 2012). Supervised PA programmes, on the other hand, appear to have some success (Dinan et al., 2006). This is not a simple intervention of course, but only certain HCPs will have the time (and willingness) to deliver it. So it cannot make every contact count as intended

Response: The Snacktivity™ intervention is not a simple verbal intervention- please see page 8 for a description of the intervention. The intervention is a behavioural change intervention with theoretical underpinnings. The Snacktivity™ intervention is based firstly on HCPs raising the topic of Snacktivity within routine consultations and thereafter the intervention involves technology support (bespoke mobile phone app and physical activity tracker that provides ongoing support to participants (page 8) for a description of the various intervention components. We agree the Making Every Contact Count initiative is a health policy initiative is reliant on HCPs being willing to deliver it and we had made this statement in the conclusion. We have re-emphasised the point again in the discussion (page 26).

It is important to advance to a quantitative study so that HCP profiles that struggle in delivery can be identified. Ideally, with patient follow-up the relative effectiveness of interventions can be studied.

Response: We will begin a phase III trial in May 2024 to test the effectiveness and cost effectiveness of the intervention within a variety of NHS settings throughout the countries of the United Kingdom. We have referred to this now on page 27.

It would also be interesting to study the effect of these interventions and the difficulties in treatment delivery on hard-to-reach populations which may be some of those most in need of PA.

Response: We agree it is important to test the intervention in hard to reach populations and this will be part of our planned effectiveness trial.

All in all, the study is well written and conducted but I expect some additional discussion on the limitations and plans for future research that will provide more concrete evidence on the matter.

Response: We would like to thank the reviewer for their kind words about our manuscript and we appreciate the feedback. We apologise the strengths and limitations section was omitted from the original manuscript but we have included this now. Please see page 27.

Minor comments

I agree that younger generations of HCP are more likely to engage in this topic but please provide a reference p.6 lines 9—11.

Response: We are sorry but we don’t understand this reviewer statement as we don’t claim that younger generations of HCPs are more likely to engage with this topic and this isn’t a view we necessarily agree with. Page 6 lines 9-11 already includes two references related to the involvement of all HCPs in delivering MECC.

p.22 line 17 It is better to write 10—11 or 10 to 11 items (10/11 resembles a proportion)

Response. Thank you - we have corrected this – see page 21.

Reviewer 2

This evaluation is a small component of what looks like a very large intervention to implement Snacktivity as part of the MECC framework across healthcare. Given the timing of the intervention, and by the authors descriptions in the text, the COVID pandemic likely had a significant effect on health care provider engagement in the study and likely the ability to collect data on patient interactions. Given the MAJOR burden shouldered by Health Care Providers (HCPs) during this timeframe, it is with this lens that primary criticisms to the robustness of the evaluation are tempered. Having said this, the major limitation of the study is the small number of HCPs evaluated and the brief exposure/experience they had with the intervention – most HCPs only implemented it with 2 patients and the most was 4, which is insufficient to make conclusions about ‘integrating’ the intervention into ‘routine’ consultations. By the authors own admission , the target intervention delivery time of 5-7 min was not achieved (10min), likely due to the HCPs’ lack of comfort delivering the intervention (i.e. most working from a script, and most acknowledging in extra comments in the end that more time to ‘work through’ or practice the intervention would have been beneficial).

Response: We agree with the reviewer comments. Due to an editing issue the strengths and limitations section was omitted from the original manuscript. We had already discussed the issue that HCPs only delivered the intervention to a small number of patients in the discussion (pages 26-27). When asking anyone to deliver a new mode of care, it will take a little time to become proficient and confident in doing so.

For these reasons, it is important for the authors to contextualize these limitations to the study, in the title, abstract and conclusions; there are also some terms that could be changed to clarify the parameters of the study. There is a lack of research on implementation of these system wide changes to practice, so the study has merit, especially the issue of implementing physical activity behaviour change into health fields that might not normally be doing lifestyle-related counselling (e.g., dentistry). The authors seemed to be hiding who the ‘health providers’ were until the methods. This can actually be reframed as a strength of the study – to really evaluate the reach of the Snacktivity within the broad framework of MECC across ALL healthcare. See suggestions below.

Response: We have tempered the abstract and conclusions are requested by the reviewer. We have also added a strength and limitations section to the discussion section of the paper. We agree that a particularly novel aspect of the study relates to implementing physical activity behaviour change into health fields that might not normally be doing lifestyle-related counselling (e.g., dentistry) and include comment in the aims and strengths of the study (page 6-7 and 27). We had highlighted in a few places that both study A and B were conducted during the COVID-19 pandemic. We have now added a comment on page 7-8 about the types of HCPs involved in the study.

Title. Recommend editing the title to more closely reflect the context of the study. Note: as the term ‘consultations’ implies that patients are coming in to discuss medical issues, whereas they may have been coming in to get a dental filling, and then had a conversation about physical activity. Health professionals’ initial experiences integrating conversations about Snacktivity™ to promote physical activity within typical medical appointments.

Response: We have elected to remain with the original manuscript title, but we have edited the manuscript to reflect the reviewer feedback, that we are presenting the initial views of HCPs.

Abstract

Study question: ‘explored’ experiences of HCPs delivering. Yes, this is accurate given that the interviews are open ended questions to explore their experience, but, there is a vast amount of literature on the challenges that HCPs face Based on previous research couldn’t’ there be a preliminary question/hypothesis?   2nd: HCP ability/fidelity to deliver Snacktivity™.

Response. We are sorry but we do not understand this review comment as we already include HCPs ability/fidelity to deliver the intervention as a second question in the abstract (and the aims of the study).

Results: ‘conceptualisation’ of Snacktivity – is this term apparently clear? This reviewer did not completely understand the intent of this term until reading through the whole paper. This is health providers’ ‘conceptualisation’.

Response. We have edited the results of the abstract to make this point regarding conceptualisation clearer and we have used the suggested wording offered by the reviewer throughout the manuscript.

‘Consultation’. Clarify as ‘health professional-patient consultation’ as it was unclear initially. This might be a language-term as medical appointments in N America are not normally referred to as ‘consultations’. ‘practical barriers to implementation’ be forthwrite with this. Although time training/education and confidence were barriers implementation.

Response. The use of the world consultation is standard terminology in the UK and Europe. We have added further detail to the abstract regarding the barriers found.

Conclusions: this sentence did not make sense until reading the whole paper. Why would developing skills in health behaviour change affect practical barriers to implementation if the fidelity was high? (i.e. they had the skill to deliver the intervention as prescribed). Its because their lack of experience with it meant it took more time than intended. So consider restating: “Health professionals were proficient and supportive of delivering the Snacktivity intervention within medical appointments. The primary barrier to implementation was the time to deliver it, however, gaining greater experience with the intervention and improving behaviour change counseling skills may reduce that barrier.”

Response. We have edited the conclusion of the abstract as suggested by the reviewer.

Strengths and limitations –

Bullet 2 – the merits of a diverse set of healthcare professionals…… in typical consultations.

Bullet 3 – in a wide range of health settings

Bullet 4 - ‘modest’ (small). consultations ‘Provider-Patient’ consultations?

Response. We have edited this section following feedback from both the Editor and reviewers.

INTRO

Good set up of the paper, although all references are UK focused. There is a large body of research from the Exercise Is Medicine initiative out of the N.America that has delineated the barriers to implementation of physical activity counselling in medical practice. Barriers such as time, education/training, confidence have all be previously shown to influence the implementation of such initiatives, so it is somewhat confusing as to why there is a need for an ‘open exploration’ of this topic. What did the researchers think they would find that is outside of the scope of the previous research on the topic?

Response. We had refer to other initiatives in addition to MECC, such as Moving Medicine and Moving Healthcare Professionals Programmes in the introduction (page 6). We had included references that refer to evidence from outside the UK (reference 1 refers to the world, reference 3 refers to Europe and reference 4 refers to Ireland). We have added some additional references that are non-UK based, including one the American initiative Exercise is Medicine (page 5). Whilst there has been some previous research regarding how to integrate conversations about health behaviour change within consultations, Snacktivity™ is a new concept and we wished to understand how HCPs were able to deliver our Snacktivity™ intervention. Very few trials of this type have recorded consultations to allow analysis of adherence to the intervention content, which is a particular strength of this study. We have strengthened reference to these points within the aims of the manuscript on page 7.

METHODS

Pg 8 line 7. HCPs across a wide diversity of health professions were recruited….. to test feasibility delivering the intervention with all areas of health services, including areas that may not normally be involved in lifestyle related behaviour change counseling such as ….((i.e. lean into this as initially it is odd that podiatry and dentistry were included in a PA intervention, but later in discussion it makes sense. So bring this intent forward).

Response: Reference to different HCPs delivering the intervention is now mentioned within the aims of the study on pages 7 and in the strengths section of the study (page 27).

The ‘intervention’ study here is not entirely clear. It is explained later (page 8 lines 3-16) Consider moving this section up front in the methods, because studies A and B evaluated here, are subsets of the Snacktivity intervention project.

Response. We have moved up the description of the intervention as suggested (page 8).

Only 2 pod delivered more than 3 consults. 5 of them 2 consults, 1-1. This is not sufficient experience with the module to make informed comments on it. References to this feedback is then 'initial experiences' not a fully informed regular practice using this intervention over several months with hundreds of patients as what would qualify 'implementation in routine care'. Authors need to change language throughout to acknowledge this.

Response: We have changed the language throughout to reflect that we are presenting HCPs initial experiences.

Quotes – somewhat representative; however, female dentist 007 and 010 compose 9 out of 23 quotes. 5 podiatrists, 3 dentists 3 physios. Is this representative of 'health care delivery' the way the MECC was intended? AS mentioned previously – it is a diverse group that would not normally be expected to deliver physical activity advice, so the authors should lean into this as a strength of testing the MECC framework.

Response: We have now included alternative quotes to make representation more balanced. We have referred to the diverse group of HCPs that were involved in the study on pages 7-8, 24 and 27.

Pg 22 – Assessing fidelity. In my opinion, following a 'script/checklist' does not display confidence/competence in delivering a counseling intervention. It typifies a lack of comfort with it, but may be required to fulfill the intervention requirements.

Response: We understand the point the reviewer is making here but in a research context it is important to include the checklist of a measure of fidelity and the HCPs were following instructions to adhere to the checklist to facilitate effective behaviour change in their patients. For HCPs, expecting them to remember to deliver every point on the checklist was unreasonable and the checklist was included as an aid to help facilitate conversations with patients. Ultimately, we do have the audio recordings which allowed us to understand these conversations in more detail.

Discussion

Pg 25 line 5 - delivering the intervention 'well' would be within the allotted time (i.e. 5 min). Delivered the intervention with 'high fidelity', although were not able to do it in the intended timeframe, possibly due to a lack of experience with behaviour change counseling and/or the intervention itself.

Response: We have edited the statement in line with the suggestion from the reviewer (page 24). Thank you.

The rest of the discussion clearly outlines the paradoxical challenge of a 'whole of health care' approach to behaviour change counseling (i.e. MECC), so this intent to 'explore' this challenge, needs to be clear up front and throughout the paper. See comments above. The rest of the discussion articulates the challenges and considerations relating to this effectively.

Response: Thank you for the supportive comments regarding the discussion. We have included a flavour of the paradoxical challenge of a whole health care approach throughout the paper.

Conclusions. Pg 28 l12 ‘some barriers’ don’t hide what these are. Be clear and forthright with it. Time and comfort/confidence.

Response: Further comments regarding barriers has been included on page 27 (conclusion).

References

Note ref 4 is to be cited as: Health Service Executive (2016). Making Every Contact Count: A Health Behaviour Change Framework and Implementation Plan for Health Professionals in the Irish Health Service. Health Service Executive. ISBN No 978-1-78602-038-3

Response: We have cited reference 4 as stated above.

VERSION 2 – REVIEW

**REVIEWER NAME**	Fowles, Jonathon
**REVIEWER AFFILIATION**	Acadia University, Kinesiology
**REVIEWER CONFLICT OF INTEREST**	JF is the Scientific Director of Exercise is Medicine Canada
**DATE REVIEW RETURNED**	05-Jun-2024

**GENERAL COMMENTS**	Authors addressed the majority of reviewer comments.
